# Sudden Death

**Published:** 1855-03

**Authors:** 


SUDDEN DEATH.—Here was a Russian on one knee, in the act of taking aim; the muzzle of his firelock rested on a forked stick. He was dead; the side of his head was knocked off by a cannon shot. His death was so sudden and quick that he was not knocked down; and the remaining part of his face still looked sternly along the firelock. It was an astonishing sight; every one that could, came to look at him.—*Extract of a letter from the Crimea.*

---

☞ During the visit of the old soldiers at Mount Vernon, while standing on the steps of the old mansion, one of the number from Ohio, aged seventy-nine, by the name of Ridgeway, said he had now living fourteen children, one hundred and ten grand-children, thirty-seven great grand-children, and seven great great grand-children, and that one of his daughters has twenty children.

